# *Phormidium ambiguum* and *Leptolyngbya ohadii* Exopolysaccharides under Low Water Availability

**DOI:** 10.3390/polym15081889

**Published:** 2023-04-14

**Authors:** Isabela C. Moia, Sara B. Pereira, Paola Domizio, Roberto De Philippis, Alessandra Adessi

**Affiliations:** 1 DAGRI—Department of Agriculture, Food, Environment and Forestry, University of Florence, Via Maragliano 77, 50144 Firenze, Italy; 2 i3S—Instituto de Investigação e Inovação em Saúde, Universidade do Porto, Rua Alfredo Allen, 208, 4200-135 Porto, Portugal; 3 IBMC—Instituto de Biologia Celular e Molecular, Universidade do Porto, Rua Alfredo Allen, 208, 4200-135 Porto, Portugal

**Keywords:** EPS monosaccharidic composition, water deprivation, soil restoration, dehydration, biocrusts, biofilms

## Abstract

Cyanobacteria can cope with various environmental stressors, due to the excretion of exopolysaccharides (EPS). However, little is known about how the composition of these polymers may change according to water availability. This work aimed at characterizing the EPS of *Phormidium ambiguum* (Oscillatoriales; Oscillatoriaceae) and *Leptolyngbya ohadii* (Pseudanabaenales; Leptolyngbyaceae), when grown as biocrusts and biofilms, subject to water deprivation. The following EPS fractions were quantified and characterized: soluble (loosely bound, LB) and condensed (tightly bound, TB) for biocrusts, released (RPS), and sheathed in *P. ambiguum* and glycocalyx (G-EPS) in *L. ohadii* for biofilms. For both cyanobacteria upon water deprivation, glucose was the main monosaccharide present and the amount of TB-EPS resulted was significantly higher, confirming its importance in these soil-based formations. Different profiles of monosaccharides composing the EPSs were observed, as for example the higher concentration of deoxysugars observed in biocrusts compared to biofilms, demonstrating the plasticity of the cells to modify EPS composition as a response to different stresses. For both cyanobacteria, both in biofilms and biocrusts, water deprivation induced the production of simpler carbohydrates, with an increased dominance index of the composing monosaccharides. The results obtained are useful in understanding how these very relevant cyanobacterial species are sensitively modifying the EPS secreted when subject to water deprivation and could lead to consider them as suitable inoculants in degraded soils.

## 1. Introduction

Cyanobacteria are a group of prokaryotic microorganisms found in fresh and marine waters and soils [1] and represent the first colonizers in drylands [2]. They have been studied for contributing to agricultural productivity in a process called “cyanobacterization”, which consists of cyanobacteria inoculation in the soil to provide soil structural stability and biofertilizer properties [3,4,5]. The selection of feasible species for a given environmental condition, allied to a cheaper biomass production and dispersion strategy, represents a new biotechnological approach to increase fertility in agricultural soils. 

This biotechnological potential is due to the formation of biocrusts, which are composed of microorganisms aggregated to soil particles [6,7]. Due to the excretion of exopolysaccharides (EPS), the biocrust-forming cyanobacteria allow: (i) the increase in soil organic carbon pool, and stimulation of exoenzyme activity in the soil [8]; (ii) water retention and infiltration, which have a positive correlation with the total carbohydrate content of the biocrust [9,10]; (iii) the retention of metabolites and nutrients, thus preventing molecules from leaching down to the subcrustal layer [11]; and (iv) the release of vitamins, amino acids and phytohormones in the soil, which act as biofertilizers [12,13]. 

The rather complex monosaccharidic composition usually provides to the cyanobacterial EPS an amphiphilic character, determining their functions in soil [14,15,16]. For example, the hydrophobic character of some fractions of the polymer, mainly due to the presence of deoxysugars, such as rhamnose and fucose, may favor the adhesion of the cyanobacterial filaments to solid substrates and the formation of soil aggregates [5,14,17]. On the other hand, the negatively charged, hydrophilic fractions of the EPS, characterized by a large presence of uronic acids and sulfate groups, are involved in binding minerals, nutrients and water molecules, thus favoring the survival of the microbial community residing in the biocrust [18,19]. These EPS features provide sustainment to the biocrusts and can enable cyanobacteria to cope with periods of desiccation [8,20,21]. 

The EPS versatility and their protection role confer resistance also for biofilms to reach ecological success [22]. For instance, the EPS hydrophobic/hydrophilic character was reported for biofilm-forming cyanobacteria isolated from wastewater treatment plant and brackish lagoon [23]. These authors reported that the high uronic acids proportions could be successfully exploited for different biotechnological applications. Moreover, Keshari et al. (2021) [24] showed that the cyanobacterium *Scytonema mille*, which has a thick sheath (polysaccharide anchored to the cell wall) was the major microorganism in the biofilms on the Buddha statue in India, indicating that the sheath may support the organism to cope with desiccation [24].

The abiotic conditions of the environment and the cyanobacterium species also influence the molecular size distribution of the EPS [9]. For example, the presence of exopolysaccharides with low molecular weight (MW) was more frequently present when the filamentous cyanobacterium *Leptolyngbya ohadii*, grown as biocrust, was subjected to high water availability [17]. This pattern might be related to the increase in the activity of enzymes that decompose the higher MW to smaller MW polymers [17]. Additionally, it was reported that *Phormidium ambiguum* liquid culture (where it experiences optimal nutritional conditions) and biocrusts produced polymers with MW distributed heterogeneously through the EPS fractions [25,26]. Therefore, the chemical and macromolecular characteristics of exopolysaccharides can be modulated according to soil type [25], nutrient supply [27], and water availability [17,21]. 

Recently, it has been shown that *L. ohadii*, isolated from biocrusts of the Negev desert [6,28], can revive after hydration/dehydration cycles [29,30]. The genus *Phormidium* has been found in arid areas, where hot deserts are present [31,32,33]. These areas are susceptible to wind erosion, due to the changes in physicochemical properties and decrease in the stability of the soil, that accelerates land degradation [34,35]. Therefore, it is important to evaluate cyanobacteria tolerating long periods of drought in order to stabilize and increase the strength of the soil. For instance, *P. ambiguum* was shown to perform an essential role in the aggregation of soil particles, improving soil structure and stability, and indicates it to be a good candidate to be used in soil restoration in arid areas [25,36]. However, little is known about these cyanobacteria growth and EPS composition and role upon low water availability when cultivated in different modes of life (sandy soil biocrusts and biofilms liquid cultures) and about the possible differences of their EPS in biofilms or biocrusts. This work aimed to characterize *P. ambiguum* and *L. ohadii* upon water deprivation in terms of the amount, monosaccharidic composition, and MW distribution of the soluble soil EPS fraction (loosely bound EPS, LB-EPS) and of the condensed soil EPS fraction (tightly bound EPS, TB-EPS) in biocrust-forming conditions, and of the RPS and anchored polysaccharides (either sheath or glycocalyx EPS) in biofilm-forming conditions. Understanding these differences could reveal the diversity of existing mechanisms to tolerate harsh environmental conditions, such as water-deprivation, determining relevant information for potential biotechnological applications.

## 2. Materials and Methods

### 2.1. Cyanobacteria Origin and Growth Conditions

Two filamentous non-heterocystous cyanobacteria, previously reported to be present in the arid soil cyanobacterial communities [30,37], were selected to evaluate the effect of water deprivation stress on their EPS secretion: the sheathed *Phormidium ambiguum* Gomont NIES-2121, purchased at NIES Collection, Japan, and originally isolated from an African soil [26], and *Leptolyngbya ohadii* provided by the Department of Plant and Environmental Sciences of the Hebrew University of Jerusalem, Israel, originally isolated in the Negev desert [30]. The latter cyanobacterium produces an EPS fraction, not structured similar to a sheath, that remains attached to the filament when grown in liquid culture hereafter referred to as glycocalyx EPS (G-EPS) [10]. Both cyanobacteria, maintained in the laboratory of the Department of Agriculture, Food, Environment, and Forestry (DAGRI) of the University of Florence (Italy), were grown in flasks containing liquid BG-11 medium [38], at 25 °C, under continuous illumination of 15 µmol photons m^−2^ s^−1^ and continuous stirring at 100 rpm until the stationary phase. These cultures were used for biomass inoculation described below.

### 2.2. Inoculation and Biocrust Sampling

In these experiments, the cyanobacterial strains were inoculated (nine replicates for each cyanobacterium) in microcosms of Petri dishes, 150 mm (diameter) × 20 mm (depth), containing 300 g of an autoclaved commercial dried silica sand (VAGA s.r.l., Pavia, Italy), with a granulometry of 0.3–0.6 mm. Before inoculation the cultures were centrifuged at different speeds to optimize their sedimentation. *P. ambiguum* was centrifuged at 7000× *g* and *L. ohadii* was centrifuged at 4000× *g*, for 20 min, at room temperature and the supernatant was discarded. To remove any remaining culture medium, the pellet filaments were resuspended in sterile distilled water, centrifuged again at the same speeds, and the supernatant discarded. The pellet was resuspended in sterile distilled water in a volume to provide enough inoculum to disperse the resuspended filaments spirally on the microcosms [10]. Each microcosm was inoculated with 160 mg (dry weight) of cyanobacterial biomass. The chlorophyll *a* amount inoculated in each microcosm corresponded to 2.54 µg/g sand and 2.9 µg/g sand for *P. ambiguum* and *L. ohadii*, respectively. Inoculated microcosms were maintained inside a Plexiglass incubator with controlled temperature at 25 °C and continuous light intensity of 30 µmol photons m^−2^ s^−1^ to stimulate growth. The microcosms were daily watered with 35 mL of sterilized distilled water (enough volume to wet the entire surface of the biocrust).

After 4 weeks, all microcosms formed a visible crust on the sand substrate and three microcosms (*N* = 3) of each cyanobacterium were randomly collected. The remaining six microcosms were kept in incubation for 5 more days under two different conditions: three (*N* = 3) were subjected to water deprivation stress by stopping the watering and leading to the drying of the biocrusts, while three (*N* = 3) the watering continued and worked as controls (Figure S1). After this additional period (totaling 33 days) the biocrusts in all six microcosms were collected. All the formed crust was collected separated from the sand substrate and gently homogenized with a sterilized spatula. After homogenization, the crusts were weighted and used to determine chlorophyll *a* content, EPS amount, monosaccharidic composition, and MW distribution as described below. 

### 2.3. Biofilm Formation in Liquid Culture Medium

Cultures of each cyanobacterium were inoculated in 1 L Pyrex Erlenmeyer flasks containing 500 mL of BG-11 culture medium [38] (nine replicates for each cyanobacterium). After inoculation, each replicate had an initial dry weight of 0.1 mg mL^−1^. The chlorophyll *a* amount inoculated in each flask corresponded to 9.65 µg mL^−1^ and 6.82 µg mL^−1^ for *P. ambiguum* and *L. ohadii*, respectively. The flasks were maintained, without agitation, inside a Plexiglass incubator with controlled temperature at 25 °C and continuous light intensity of 30 µmol photons m^−2^ s^−1^ to stimulate growth. After 4 weeks, 3 biofilms trials (*N* = 3) of each cyanobacterium were randomly collected. The remaining 6 biofilms trials were kept in incubation for 5 more days, under two different conditions: 3 (*N* = 3) were subjected to water deprivation stress by removing the liquid medium and maintaining the biofilm attached to the glass bottom of the Erlenmeyer flasks, while 3 (*N* = 3) were kept with the liquid medium (Figure S1). After this additional period (totaling 33 days) the 6 biofilms were collected. Samples were used to determine chlorophyll *a* content, EPS amount, monosaccharidic composition, and MW distribution as described below. 

### 2.4. Biocrusts and Biofilm Characterization

#### 2.4.1. Growth Measurements through Chlorophyll *a*

For biocrusts, chlorophyll *a* content was determined according to the method as reported in Castle et al. (2011) [39]. All biofilms, after resuspension of the water deprived samples in saline solution (150 mM NaCl in sterile distilled water), were homogenized with a sterilized glass rod and collected with serological pipette. The extraction was performed according to the method as reported in Ritchie (2006) and Yéprémian et al. 2016 [40,41]. Briefly, 1 g of homogenized crust and 2 mL of biofilm homogenate were collected into screw-cap vials. The biofilms were centrifuged at 3800× *g* for 10 min, and the supernatant discarded. Next, the weighted crust and the pelletized biofilm were treated with 5 mL of ethanol, at 80 °C, for 5 min. Samples were incubated in the dark, at 4 °C for 30 min before being centrifuged at 3800× *g* for 15 min. The supernatant was recovered and determined by measuring the absorbance (A) at 665 nm. Chlorophyll *a* content was calculated according to previous study [40]:

Chlorophyll *a* [µg/g crust] = (11.9035 × A_665_ × Ve) × (g *crust*^−1^) × L (1)

Chlorophyll *a* [µg/mL] = (11.9035 × A_665_ × Ve) × (Vs^−1^) × L (2)

where Ve is the volume of ethanol (mL), Vs is the volume of sample (mL), and L is the path length (cm). 

#### 2.4.2. EPS Isolation, Quantification and Characterization

The EPSs were extracted from the biocrusts in two different fractions according to previous works [17,19,42]. The one easily released into the sand substrate and more water soluble, referred to as loosely bound EPS (LB-EPS), and the one more condensed, firmly attached to cells and sand particles, referred to as tightly bound EPS (TB-EPS). LB-EPS were recovered by resuspending the biocrusts in distilled water and incubating them at room temperature for 15 min. Next, samples were centrifuged at 3800× *g* at 8 °C for 30 min and the LB-EPS-containing supernatants collected. This extraction was repeated three times for each sample. TB-EPS were recovered by treating the biocrust pellet resulting from the LB-EPS extraction with 0.1 M Na_2_EDTA for 16 h at room temperature. Next, samples were centrifuged at 3800× *g* at 8 °C for 30 min and the TB-EPS-containing supernatants collected. This extraction was repeated three times for each sample, the last two extractions performed for 120 min each. 

Regarding the biofilms, EPS were extracted from the glass-rod homogenized biofilms cultures and centrifuged at 3800× *g* for 30 min at room temperature. The RPS were isolated from the supernatant while the pelleted cells were saved for sheath or G-EPS extraction. For that, the supernatants were concentrated by evaporation using an orbital evaporator at 35 °C. Next, the RPS were precipitated by mixing with two volumes of cold (4 °C) isopropyl alcohol and incubated at 4 °C for 8 h. After centrifuging at 4000× *g* for 15 min, the EPS pellets were resuspended in distilled water. For sheath or G-EPS extraction, the pelleted cyanobacterial cells obtained after the centrifugation of the culture biofilms were washed with 5 mL of 1.5% NaCl solution. After removing the 1.5% NaCl solution, the pellets were resuspended in 5 mL of sterile distilled water at 80 °C for 1 h [19]. After centrifuging at 4000× *g* for 30 min, the sheath-containing supernatants for *P. ambiguum* and the G-EPS for *L. ohadii* were collected. 

The RPS and TB-EPS extracts were confined in dialysis membranes (12–14 kDa MW cut off, Medicell International London) and dialyzed against distilled water for 24 h, with two changes of water. All the EPS extracts were quantified by the phenol-sulphuric acid assay method [43]. For the determination of the monosaccharidic composition, the samples were hydrolyzed in 2 N trifluoroacetic acid (TFA), for 120 min at 120 °C. The tubes containing the samples were then dipped in cool water and the samples were evaporated in an orbital evaporator at 35 °C. Subsequently, samples were suspended in HPLC-grade water and evaporated again, repeating this step for one more time. Finally, monosaccharide composition was analyzed with a Dionex ICS-2500 ion exchange chromatograph (Dionex, United States) equipped with an ED50 pulsed amperometric detector operating with a gold working electrode (Dionex) and a CarboPac PA1 column of 250 mm length and 4.6 mm internal diameter (Dionex, Sunnyvale, CA, USA). Eluents were HPLC-grade water (A), 0.185 M NaOH (B), and 0.488 M sodium acetate (C), at a flow rate of 1 mL min^−1^. Single sugars were identified and quantified based on the retention time of reference standards. Results were expressed in molar ratio.

Molecular size distribution of the EPSs was analyzed using a Varian ProStar HPLC chromatograph (Varian, CA, USA) equipped with a refractive index detector and two columns for Size Exclusion Chromatography (Polysep-GFC-P6000 and 4000, Phenomenex, CA, USA) connected in series. Samples were analyzed with runs of 70 min and with HPLC-grade water as eluent, at a flow rate of 0.4 mL min^−1^. Dextran (Sigma-Aldrich, Burlington, MA, USA) at different MWs (2, 1.1, 0.41, 0.15, and 0.05 M Da) were used as reference standards. To obtain the % of the different MW classes the ratio between each peak area and the total area under the curve was calculated and the resulting % area was assigned to the corresponding size class according to the retention time of the peak output. 

### 2.5. Data Analysis

Possible differences in chlorophyll *a* and EPSs amount among the two cyanobacterial strains grown in biocrusts and biofilm-cultures were analyzed using one-way analysis of the variance (ANOVA) at 95% of the significance, followed by Tukey post hoc test. Variables were previously checked for normality and homogeneity of variance using the Shapiro–Wilk and Levene’s test, respectively. To correlate parameters, linear regression analyses were performed, and *r*^2^ and *p* values are reported. For statistical analysis of the monosaccharidic profiles, Student’s *t* test was used to compare the relative amount of each monosaccharide in the control and water deprivation replicates. Furthermore, the number of monosaccharides, diversity, dominance, and equitability indices of sugar residues of the different strains and conditions (sheath, G-EPS, and RPS in liquid cultures, and LB-EPS and TB-EPS in biocrusts) were compared. Shannon diversity index was calculated using the percentiles of a bootstrap distribution with 9999 repetitions. All statistical analysis was performed using Past 4.09 software. 

## 3. Results

### 3.1. Effect of Water Deprivation on Chlorophyll a and EPS Production in Sandy Microcosms

**Figure 1:**
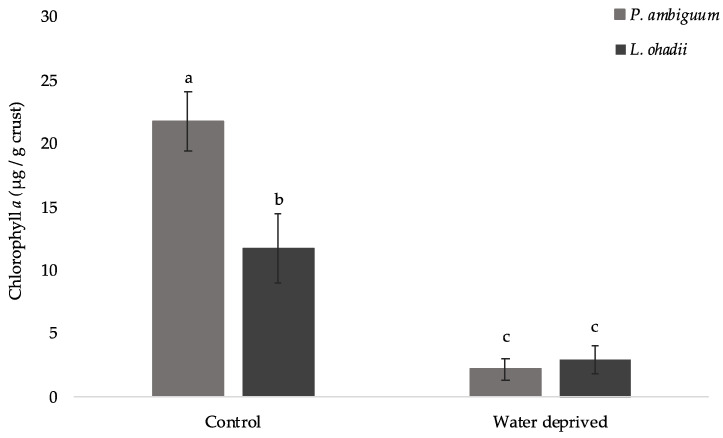
Chlorophyll *a* content in cyanobacterial biocrusts (values represent the mean of *N* = 3, error bars represent SD). Different letters represent significant differences (*p* < 0.05).

**Figure 2:**
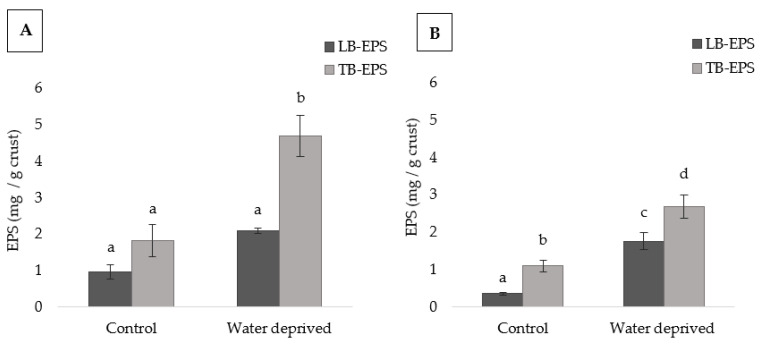
EPSs contents in *P. ambiguum* (**A**) and *L. ohadii* (**B**) biocrusts (values represent the mean of *N* = 3, error bars represent SD). Different letters represent significant differences (*p* < 0.05) in each graph.

The growth of the cyanobacterial strains was evaluated by measuring the chlorophyll *a* content. Chlorophyll *a* content decreased significantly (*p* < 0.05) during the water deprivation period for both cyanobacteria (Figure 1). The content of chlorophyll *a* decreased in a negative correlation with the EPSs contents (Figure 2), presenting an r^2^ of 0.87, 0.84, 0.89, and 0.77, respectively; *p* < 0.05, for *P. ambiguum* LB-EPS, *P. ambiguum* TB-EPS, *L. ohadii* LB-EPS, and *L. ohadii* TB-EPS, respectively. The significantly higher amount of chlorophyll a obtained for *P. ambiguum* control (i.e., 4 weeks watered plus 5 days watered), represented twice the amount of chlorophyll *a* produced by *L. ohadii* control (Figure 1). 

The amount of LB-EPS, the loosely bound polymers, that are weakly attached to cells and sediments, and the TB-EPS, the tightly bound polymers, which have stronger bounds to cells and sediments, changed according to the cyanobacterial strain and growth condition (Figure 2 and Figure S2). For *P. ambiguum*, the only significant difference observed was the increase in the TB-EPS fraction in water deprivation compared to the TB-EPS from the control and all the LB-EPS fractions. On the other hand, for *L. ohadii,* in LB-EPS from water deprived condition was significantly higher compared to those of the control. For this strain, the TB-EPS amount was significantly higher than the LB-EPS amount in each condition. The TB-EPS after water deprivation resulted significantly the highest value among all the EPSs extracted for both cyanobacteria (Figure 2).

When comparing the amount of LB-EPS in each condition for both cyanobacteria, those from *P. ambiguum* in control showed significantly higher value than those of *L. ohadii* in the same condition (Figure S3). After the water deprivation period, the LB-EPSs of these strains were not statistically different. On the contrary, while *P. ambiguum* TB-EPS control was not statistically different from *L. ohadii* TB-EPS control, after the water deprivation period, *P. ambiguum* TB-EPS showed significantly higher value than *L. ohadii* TB-EPS. 

### 3.2. Changes in Monosaccharidic Composition and Molecular Weight Distribution of Microcosms EPSs

**Figure 3:**
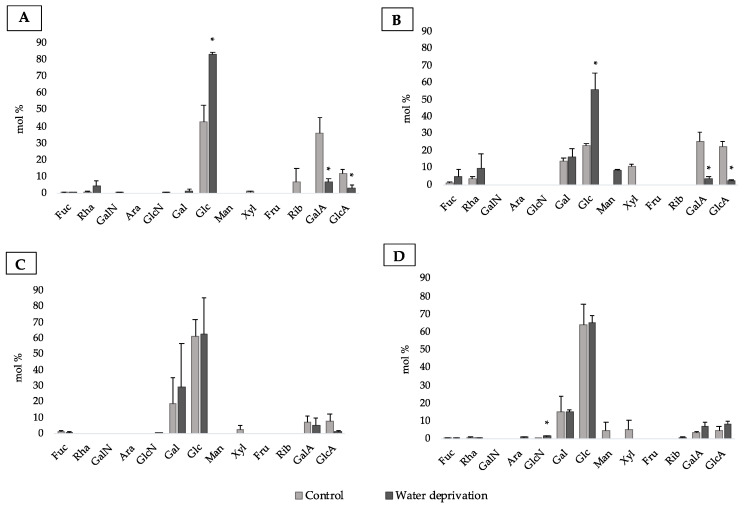
Monosaccharidic composition of the EPS extracted from biocrusts: (**A**) LB-EPS from *P. ambiguum,* (**B**) LB-EPS from *L. ohadii*, (**C**) TB-EPS from *P. ambiguum*, (**D**) TB-EPS from *L. ohadii*. Molar percentages (%) of single sugars are represented (expressed as moles of the single monosaccharide divided by the total amount of moles of monosaccharides in the EPS × 100). Symbol *, when present, indicates significant differences between the control and watered-deprived period in each monosaccharide. Fuc, fucose; Rha, rhamnose; GalN, galactosamine; Ara, arabinose; GlcN, glucosamine; Gal, galactose; Glc, glucose; Man, mannose; Xyl, xylose; Fru, fructose; Rib, ribose; GalA, galacturonic acid; GlcA, glucuronic acid.

The EPSs were analyzed in terms of monosaccharide composition, showing differences in its relative abundances between the control and water deprived microcosms. The EPS fractions of *P. ambiguum* were mainly composed of glucose (Figure 3 and Figure S2). The LB-EPS after water deprivation had the highest relative amount of this sugar among the EPSs extracted from this cyanobacterium biocrust. On the other hand, the LB-EPS in the control microcosms had a higher diversity and was also composed by significantly higher molar ratios of uronic acids (Figure 3A,C and Table S1). Moreover, fucose and rhamnose were detected in LB-EPS control and water deprived. The TB-EPS of water deprived *P. ambiguum* microcosms showed lower diversity than the control TB-EPS of this cyanobacterium (Table S1). When comparing the LB-EPS and TB-EPS only in water deprivation conditions, the first fraction showed lower diversity than the latter (Table S1). Galactose was found only after water deprivation in LB-EPS and in both conditions in TB-EPS (Figure 3A,C). Additionally, fucose was detected in the control and water deprived TB-EPS.

After 4 weeks watered, *L. ohadii* LB-EPS and TB-EPS were mainly composed of glucose (Figure S2). The control LB-EPS and TB-EPS were mainly composed of glucose, galactose, and uronic acids (Figure 3B,D). The control TB-EPS showed higher diversity than the TB-EPS extracted after water deprivation (Table S1). For this cyanobacterium, the TB-EPS of water-deprived microcosms showed the highest molar percentage of glucose, followed by galactose. This EPS fraction after water deprivation condition had higher dominance than the control TB-EPS, the same profile observed for the LB-EPS. When comparing the LB-EPS and TB-EPS only in water deprivation conditions, the first fraction showed higher diversity than the second fraction. The TB-EPS demonstrated higher dominance compared with LB-EPS, the same profile shown by *P. ambiguum* water-deprived biocrusts. Moreover, fucose and rhamnose were present in both control and water-deprived EPS fractions. 

All the EPS extracted were analyzed in terms of molecular weight (MW) distribution. The results revealed the presence of molecules ranging from 50 kDa to 2 MDa. The *P. ambiguum* LB-EPS and TB-EPS were composed predominantly of MW molecules of 410 kDa, except LB-EPS in water deprivation stress that also showed molecules higher than 2 MDa. The *L. ohadii* LB-EPS after the watered periods was predominantly composed by molecular weight molecules of 1.1 MDa–410 kDa and after a water deprivation of 1.1 MDa. The TB-EPSs extracted from this cyanobacterium were mainly constituted of molecules with MW ranging between 2 MDa–1.1 MDa (80% of chromatogram area) and a small fraction of molecules with MW ranging between 410 kDa–150 kDa (15% of chromatogram area) for all the conditions tested (data not shown).

### 3.3. Effects of Water Deprivation in Chlorophyll a and EPS Production in Biofilms

**Figure 4:**
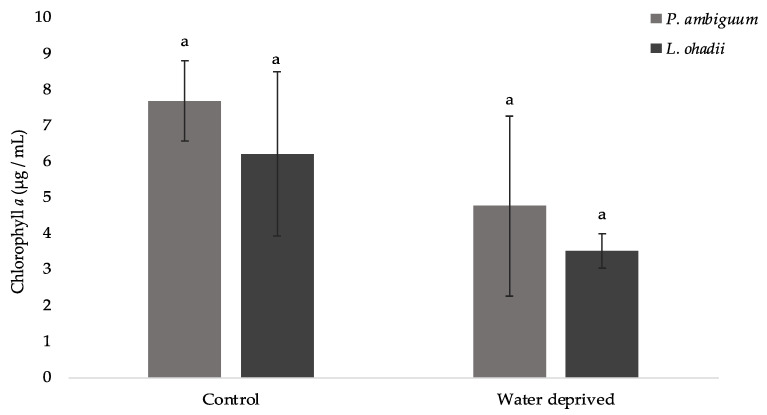
Chlorophyll *a* content in cyanobacterial biofilms (values represent the mean of *N* = 3, error bars represent SD). Different letters represent significant differences (*p* < 0.05).

For the controls and the water-deprived biofilms, no cyanobacterium produced significantly more chlorophyll *a* than the other (Figure 4). Therefore, no significant correlation could be attributed between the EPSs contents and chlorophyll a (*p* > 0.05).

**Figure 5:**
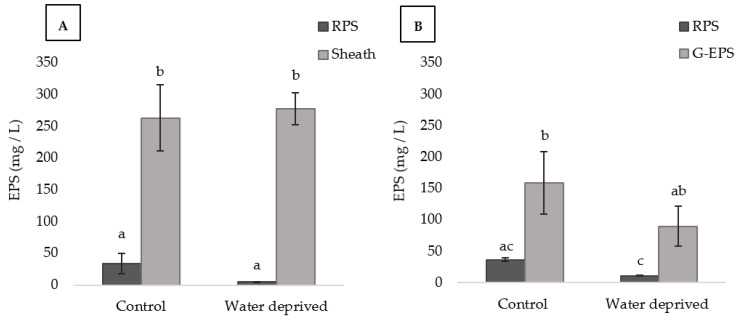
EPSs contents in *P. ambiguum* (**A**) and *L. ohadii* (**B**) biofilms (values represent the mean of *N* = 3, error bars represent SD). Different letters represent significant differences in each graph (*p* < 0.05).

For both cyanobacteria, the amount of RPS and sheath or G-EPS did not show significant differences between the control and the water-deprived biofilms (Figure 5A,B). For *P. ambiguum*, the sheath EPS amount was significantly higher, under all conditions, than the RPS, indicating that the sheath gives the major contribution to the total carbohydrates of this cyanobacterium (Figure 5A and Figure S4B) in biofilm forming conditions. For *L. ohadii* under water deprivation stress, the G-EPS was significantly higher than the RPS, but it was not significantly higher than the control RPS (Figure 5B). Moreover, when comparing the RPS between the cyanobacteria in each condition, *L. ohadii* RPS after water deprivation stress resulted significantly higher (Figure S5A). On the other hand, the sheath EPS resulted significantly higher than G-EPS in water deprivation stress, indicating how sheath EPS could contribute to *P. ambiguum* as protection in biofilms (Figure S5B). 

### 3.4. Changes in Monosaccharidic Composition and Molecular Weight Distribution of Biofilms EPSs

**Figure 6:**
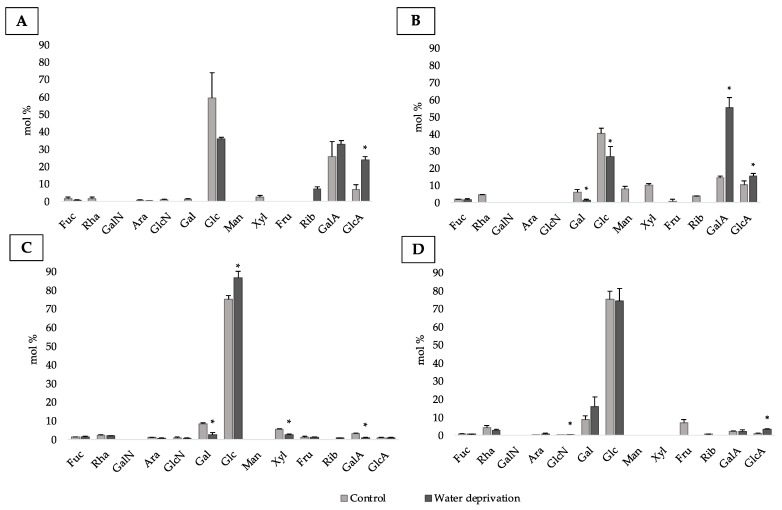
Monosaccharidic composition of the EPS extracted from biofilms: (**A**) RPS from *P. ambiguum*, (**B**) RPS from *L. ohadii*, (**C**) Sheath EPS, (**D**) G-EPS. Molar percentages (%) of single sugars are represented (expressed as moles of the single monosaccharide divided by the total amount of moles of monosaccharides in the EPS × 100). Symbol *, when present, indicates significant differences between the control and the water-deprived period in each monosaccharide. Fuc, fucose; Rha, rhamnose; GalN, galactosamine; Ara, arabinose; GlcN, glucosamine; Gal, galactose; Glc, glucose; Man, mannose; Xyl, xylose; Fru, fructose; Rib, ribose; GalA, galacturonic acid; GlcA, glucuronic acid.

The monosaccharidic composition revealed differences in biofilm-cultures, showing different profiles between the strains, the conditions, and comparing them to the biocrusts. The RPS after water deprivation were mainly composed of glucose and uronic acids (Figure 6A,B). After water deprivation, only glucuronic acid demonstrated significantly higher molar ratio than the control for *P. ambiguum* RPS. Unlike *P. ambiguum* LB-EPS, the molar ratio of glucose in *P. ambiguum* RPS did not show significant differences between the control and water deprivation. The RPS after water deprivation showed higher equitability than the RPSs extracted from the control biofilms, conferring the highest diversity, despite the smaller number of monosaccharides (Table S2). The sheath EPS after water deprivation showed lower diversity than the RPS after water deprivation and the sheath EPS control, due to the higher dominance of glucose. This moiety also demonstrated significantly higher molar ratio than the control in the sheath EPS (Figure 6C, Table S2). 

For all the conditions, the RPSs demonstrated higher diversity than the G-EPS in *L. ohadii* biofilm (Table S2). For this cyanobacterium RPS, the galacturonic and glucuronic acids were significantly higher in water deprivation compared to the control. A similar profile was found in *P. ambiguum* biofilm, in which these sugars dominated the RPS after water deprivation. Unlike *L. ohadii* LB-EPS, the molar ratio of glucose in *L. ohadii* RPS after water deprivation was significantly lower compared to the control. In addition, unlike *P. ambiguum* biofilm, the *L. ohadii* RPS in water deprivation showed lower diversity compared with the RPS control. The latter resulted in the highest number of monosaccharides and the highest equitability, leading to the highest diversity among all the *L. ohadii* EPS fractions. Finally, glucose and galactose were the monosaccharides mostly present in G-EPSs (Figure 6D). The G-EPS after water deprivation showed lower diversity than the RPS after water deprivation and the G-EPS control, due to the higher dominance of glucose as observed in the sheath EPS after water deprivation (Figure 6B–D, Table S2). 

The EPS extracted from biofilms were analyzed in terms of molecular weight (MW) distribution. The MW distribution of the RPSs extracted were not detectable and data are not shown. The sheath EPSs of *P. ambiguum* were predominantly composed of molecules lower than 50 kDa, while the *L. ohadii* G-EPSs were constituted of molecules having an apparent MW between 410 kDa and 50 kDa (data not shown). 

## 4. Discussion

Filamentous cyanobacteria have been studied for their potential to survive water deprivation [29,44,45]. Previous studies demonstrated that the profile of exopolysaccharides is heterogeneously dependent on the strain [17,26,42]. In this study, two different strains were cultivated as biocrusts and biofilms, showing different responses to water deprivation stress. 

### 4.1. EPS and Growth Profile in Biocrust-Forming Cyanobacteria

When the two cyanobacteria were incubated in microcosms, the amount of chlorophyll *a* decreased under water deprivation, indicating that five days deprivation of water had an impact in photosynthesis. The photosynthetic apparatus has been reported to respond to water deprivation conditions [30,31,46,47,48]. These authors reported that the phycobilisomes in the desiccated state lose their organized structure, and after re-wetting, photosynthetic activity was reactivated. Previous study identified genes encoding proteins involved in photosynthesis and chlorophyll *a* synthesis whose transcription was induced by re-wetting the biocrust of *Microcoleus vaginatus* [49]. Additionally, previous work reported that the dry phase induces an osmotic stress, which regulates genes and decreases the photosynthesis activity. Therefore, this agrees with our observation of a decrease in chlorophyll *a* induced by water deprivation [50,51]. 

Cultures of bacteria and cyanobacteria have been reported to increase EPS production under water deprived conditions, suggesting that resources were allocated to EPS production in response to the stress [52,53,54]. This may explain the apparent contradiction between the increase in EPS and the decrease in photosynthesis. In this work, the amount of the EPS changed with the water availability: *P. ambiguum* TB-EPS amount was significantly higher only under water deprivation. It was previously reported that this cyanobacterium promotes higher increase in TB-EPS compared to other cyanobacteria (i.e., *Scytonema javanicum*) [26]. In the present work, it also promoted significantly higher TB-EPS amounts compared to *L. ohadii* biocrusts under water deprivation (Figure S3B), corroborating with previously reports that *P. ambiguum* has a more prominent role of aggregation of soil particles and improvement of soil strength, due to the synthesis of large amounts of TB-EPS [25,26,36]. This could be an advantage for this cyanobacterium to deal with periods of limited water availability. The significant increase in the amount of LB-EPS of *L. ohadii* under the water deprivation stress condition corroborates previous findings [17] that the amount of LB-EPS in *L. ohadii* biocrusts increases when incubated with lower amounts of water. This suggests that the amount of water influences the amount of LB-EPS for this cyanobacterium. On the other hand, in contrast to previous reports that the amount of *L. ohadii* TB-EPS was not influenced by water availability, in the present study the amount of TB-EPS was higher under water deprivation condition, being significantly higher than the other EPSs fractions quantified for *L. ohadii* biocrust justifying their large presence in biocrusts that are subject to frequent dry and wet cycles [55].

Not only the amount of EPSs can be modulated by the harsh environmental conditions to which cyanobacteria were submitted, but also their monosaccharidic composition [8]. The higher relative abundance of glucose in the LB-EPS and TB-EPS of both cyanobacterial biocrusts, and the increase in the molar ratio of this sugar in water deprivation conditions is in agreement with previous studies that reported the higher abundance of glucose in sand biocrusts of these cyanobacteria under low water additions, mainly in LB-EPS [17,25,26]. Moreover, galactose was another sugar found in biocrusts that present higher molar ratios compared to other sugars. It has been reported to be highly produced by *P. ambiguum* TB-EPS in sand soils [25], confirming the observations on *P. ambiguum* biocrusts reported here. These data corroborate previous findings that, in nutrient limited conditions often found in biocrusts from drylands, more essential monosaccharides are required by the cyanobacteria [56], which could explain the increase in the dominance index in water deprivation stress condition. 

Despite the dominance of glucose, cyanobacterial EPS are also characterized by the presence of uronic acids and deoxysugars, such as fucose and rhamnose [57,58,59]. In this work, uronic acids were present in significantly higher percentage after the watered periods and decreased after the water deprivation period. The uronic acids have hydrophilic character involved in the chelation of minerals, nutrients, and water molecules when present [18] and have been detected mostly under the highest water availability in *L. ohadii* LB-EPS [17]. On the contrary, fucose and rhamnose have hydrophobic character increasing cell capacity to adhere to solid surfaces [5,19]. Though the mol% of these monosaccharides is probably too low, in our results, to observe a significant difference in polymer hydrophobicity, these data still show the plasticity of the cells to modify EPSs to respond to the surrounding environment performing with the amphiphilic character of the macromolecules. In addition, the sugar amount and composition were different between the strains and EPS fractions, showing how these polymers are heterogeneously dependent on the cyanobacteria and abiotic factors. 

The molecular weight distribution of EPS is another feature that deserves further investigations about its response to water deprivation. In this work, the TB-EPSs extracted were mainly composed of molecules ranging between 2 MDa–410 kDa of apparent MW. The biocrust stability was previously reported to be provided by the TB-EPS, which was composed of MW in the range 410 kDa–50 kDa, but by higher abundance of MW in the range 2 MDa–1.1 MDa [25,26,36]. Moreover, the LB-EPSs were also represented by >2 MDa–1.1 MDa molecules after water deprivation, while after the watered periods molecules in the range 1.1 MDa–410 kDa were present. The presence of high MW molecules could also be due to molecule aggregation after the extraction. However, the analysis confirms the presence of large polymers, which can contribute to the viscosity of the EPS. The presence of such high MW molecules under low moisture could indicate a higher water retention during periods of drought [17,25,60]. 

### 4.2. Biocrusts and Biofilms EPSs, Similarities and Differences

The sheath EPS is important for mechanical and physicochemical stability [61,62,63]. On stones and monuments, the presence of biofilm-forming sheathed cyanobacteria has already been reported [18,64]. The thick sheath protects the cyanobacteria, helping it to survive in low nutrient environment [22], high temperatures, and water deprivation conditions [24,63]. This EPS structure has been documented to be thicker in stressed cells [65] and strongly connects sand grains [60,66]. Taking that into consideration, it is possible that the *P. ambiguum* TB-EPS might be similar to the sheath EPS, due to the similar role performed by the two EPS fractions. These findings were also reported by previous work which documented the relation between TB-EPS and sheath EPS produced by Schizothrix cf. delicatissima [42]. Regarding the *L. ohadii* TB-EPS and G-EPS some similarities in the monosaccharidic composition were observed. Both fractions have predominantly abundances of glucose and galactose that were not significantly different between the control and water-deprived samples. This could indicate that also the TB-EPS might derive from the G-EPS, as suggested by previous authors who reported that the dominant presence of glucose in *L. ohadii* G-EPS combined with other sugars are related with stronger sand stabilizing capability [10], a feature related to TB-EPS [8,19]. 

In sand biocrusts, the LB-EPS is released and remains in the biocrusts, with no loss of polysaccharides as in water-deprived biofilms. Furthermore, after water deprivation, rhamnose was detected in both cyanobacteria LB-EPSs, but not in the RPSs, showing the relevance of this hydrophobic monosaccharide in water-deprived biocrusts. This is particularly important for demonstrating the functionality of LB-EPS in contributing to surface hydrophobicity [5]. The presence of uronic acids in *P. ambiguum* and *L. ohadii* RPS was already observed by previous studies [10,26], but here it was demonstrated that even in water deprivation these sugars are predominantly present. In this stressful condition, the RPS fraction from the cyanobacteria tested in this work showed a different relative amount of these sugars from the LB-EPSs. While in the latter significantly increased the amount of glucose and reduced the amount of uronic acids, in the RPSs the amount of glucose significantly decreased while the amount of uronic acids increased compared to their controls. This suggests that the water-deprived condition for biofilms may stimulate intracellular enzymes that oxidize glucose to form glucuronic acid, which in turn is converted to galacturonic acid [67,68,69], since the cyanobacteria may be undergoing an oxidation process imposed by the absence of culture medium [70,71,72,73]. This behavior shows how the environment can modulate the monosaccharidic profile. Therefore, without the sand substrate, the relative abundance of the sugars changed after water deprivation. Moreover, the diverse composition of monosaccharides and MW distribution between the cyanobacteria supports the existence of different response to the surrounding environment in normal or under stress conditions [74].

## 5. Conclusions

The present work provided important indications regarding the defense mechanisms of two cyanobacteria, namely, *P. ambiguum* and *L. ohadii,* to water deprivation in biocrusts and in biofilm formation. The strains showed EPS synthesis capability even in water deprived conditions, demonstrating a distinct production pattern of EPS. Particularly the production of TB-EPS was significantly increased in biocrusts after the water deprivation, suggesting the importance of this fraction in slowing down desiccation. In biofilms under water deprivation, the sheath EPS was significantly more abundant than the RPSs and G-EPS, suggesting that it may perform a crucial role in stress tolerance. The different profile of monosaccharidic composition demonstrated the plasticity of the cells to modify EPS in order to respond different stresses, as reported here by the higher concentration of deoxysugars in biocrusts compared to biofilms. For the two cyanobacteria evaluated in this work, water-deprived biofilms and biocrusts tend to produce simpler carbohydrates and increase their dominance index. The knowledge of different cyanobacteria biological mechanisms to cope with harsh environmental conditions is important to select the best strain for the use in the restoration of dryland soils and the improvement of ecosystems services.

## Data Availability

Not applicable.

## Supplementary Materials

The following supporting information can be downloaded at: https://www.mdpi.com/article/10.3390/polym15081889/s1, Figure S1: biocrusts and biofilms control and water deprived; Figure S2: biocrust features after 4 weeks watered; Figure S3: LB-EPS and TB-EPS contents ; Figure S4: biofilm features after 4 weeks with culture medium; Figure S5: RPS, sheath and G-EPS contents; Table S1: diversity indices of the biocrusts EPS; Table S2: diversity indices of the biofilms EPS; Table S3: sugar total mols and mols of each monosaccharide in each EPS fraction for *P. ambiguum* biocrusts; Table S4: sugar total mols and mols of each monosaccharide in each EPS fraction for *L. ohadii* biocrusts; Table S5: sugar total mols and mols of each monosaccharide in each EPS fraction for *P. ambiguum* biofilms; Table S6: sugar total mols and mols of each monosaccharide in each EPS fraction for *L. ohadii* biofilms.
