# Supplementary material for: Phormidium ambiguum and Leptolyngbya ohadii Exopolysaccharides under Low Water Availability
Source: Polymers (Basel). 2023 Apr 14;15(8):1889. doi: 10.3390/polym15081889 (PMC10142279; doi:10.3390/polym15081889)
Supplement: Supplementary file 1 [file polymers-15-01889-s001.zip › polymers-2213156-supplementary.pdf]

Supplementary Materials

Phormidium ambiguum and *Leptolyngbya ohadii* Exopolysaccharides under Low Water Availability

Isabela C. Moia[1], Sara B. Pereira[2,3], Paola Domizio[1], Roberto De Philippis[1], Alessandra Adessi[1]*

[1] DAGRI - Department of Agriculture, Food, Environment and Forestry, University of Florence, Via Maragliano 77, 50144 Firenze, Italy

[2] i3S - Instituto de Investigação e Inovação em Saúde, Universidade do Porto, Rua Alfredo Allen, 208, 4200-135 Porto, Portugal

[3] IBMC - Instituto de Biologia Celular e Molecular, Universidade do Porto, Rua Alfredo Allen, 208, 4200-135 Porto, Portugal

* Correspondence: alessandra.adessi@unifi.it

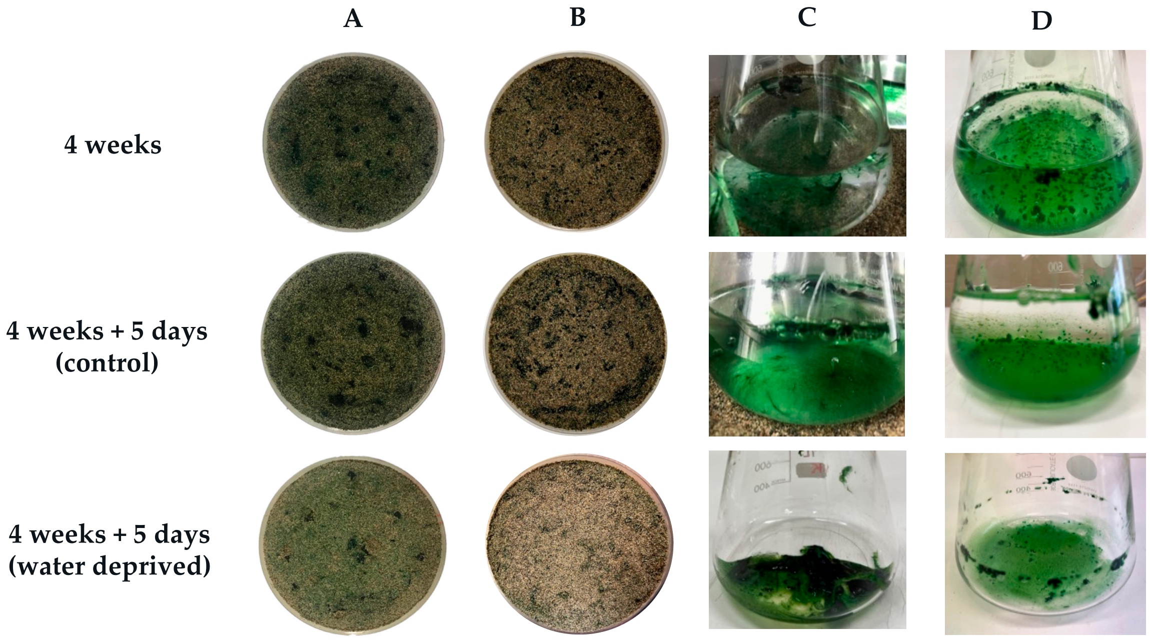

Figure S1. Biocrusts and biofilms grown after 4 weeks and 4 weeks plus 5 days (control and water deprived). *P. ambiguum* (column A) and *L. ohadii* (column B) biocrusts and *P. ambiguum* (column C) and *L. ohadii* (column D) biofilms.

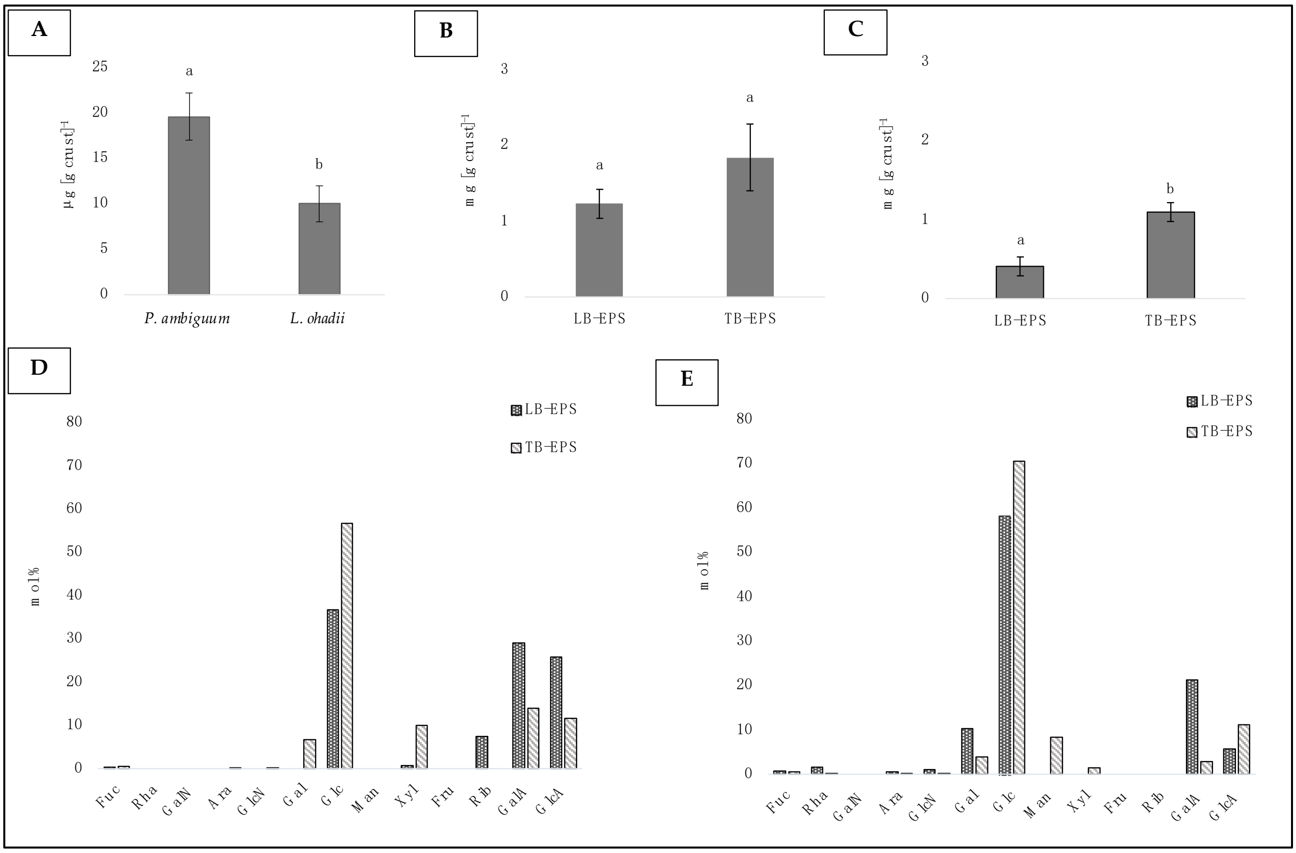

Figure S2. Biocrust features after 4 weeks watered: (A) chlorophyll *a*, (B) EPS contents from *P. ambiguum*, (C) EPS contents from *L. ohadii*, (D) monosaccharidic composition from *P. ambiguum*, (E) monosaccharidic composition from *L. ohadii*. Values represent the mean of $N = 3$, error bars represent SD. Different letters represent significant differences ($P < 0.05$). Molar percentages (%) of single sugars are represented (expressed as moles of the single monosaccharide divided by the total amount of moles of monosaccharides in the EPS × 100). Fuc, fucose; Rha, rhamnose; GalN, galactosamine; Ara, arabinose; GlcN, glucosamine; Gal, galactose; Glc, glucose; Man, mannose; Xyl, xylose; Fru, fructose; Rib, ribose; GalA, galacturonic acid; GlcA, glucuronic acid.

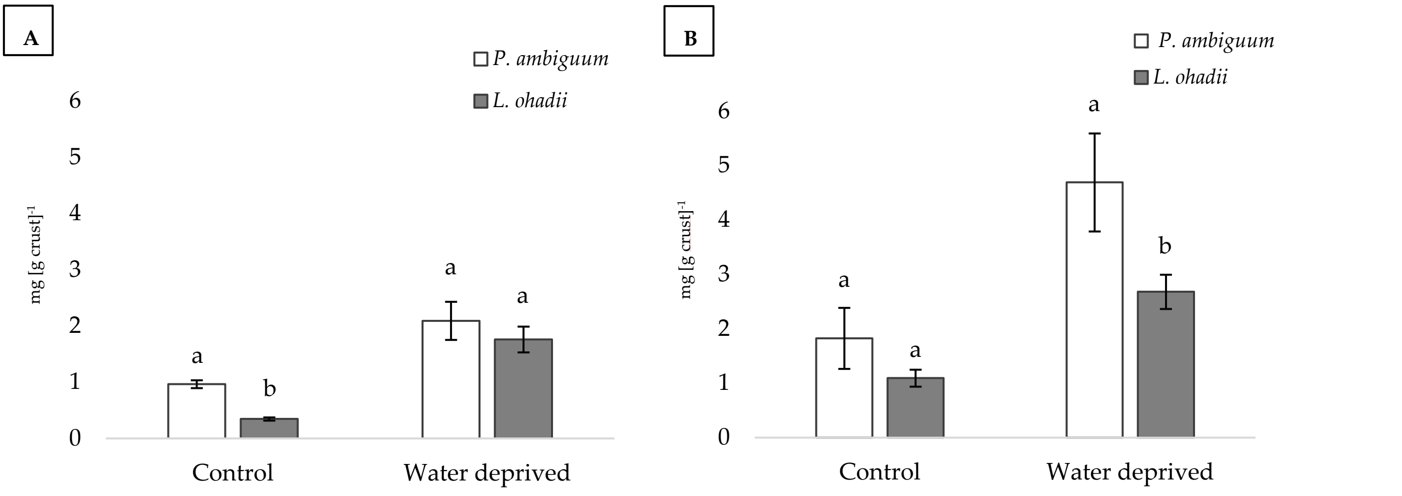

Figure S3. LB-EPSs contents (A) and TB-EPSs contents (B) in *P. ambiguum* and *L. ohadii* biocrusts (values represent the mean of $N = 3$, error bars represent SD). Different letters represent significant differences ($P < 0.05$) in each condition.

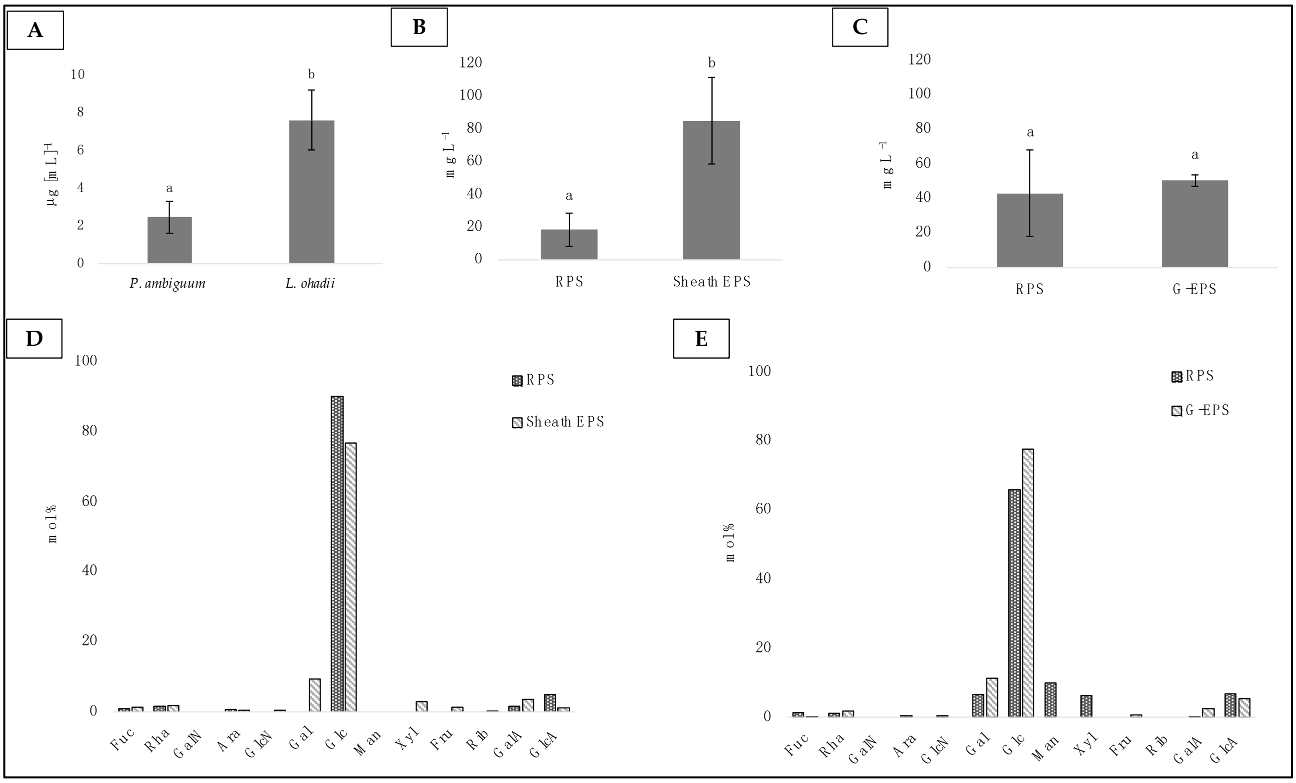

Figure S4. Biofilm features after 4 weeks with culture medium: (A) chlorophyll *a*, (B) EPS contents from *P. ambiguum*, (C) EPS contents from *L. ohadii*, (D) monosaccharidic composition from *P. ambiguum*, (E) monosaccharidic composition from *L. ohadii*. Values represent the mean of $N = 3$, error bars represent SD. Different letters represent significant differences ($P < 0.05$). Molar percentages (%) of single sugars are represented (expressed as moles of the single monosaccharide divided by the total amount of moles of monosaccharides in the EPS × 100). Fuc, fucose; Rha, rhamnose; GalN, galactosamine; Ara, arabinose; GlcN, glucosamine; Gal, galactose; Glc, glucose; Man, mannose; Xyl, xylose; Fru, fructose; Rib, ribose; GalA, galacturonic acid; GlcA, glucuronic acid.

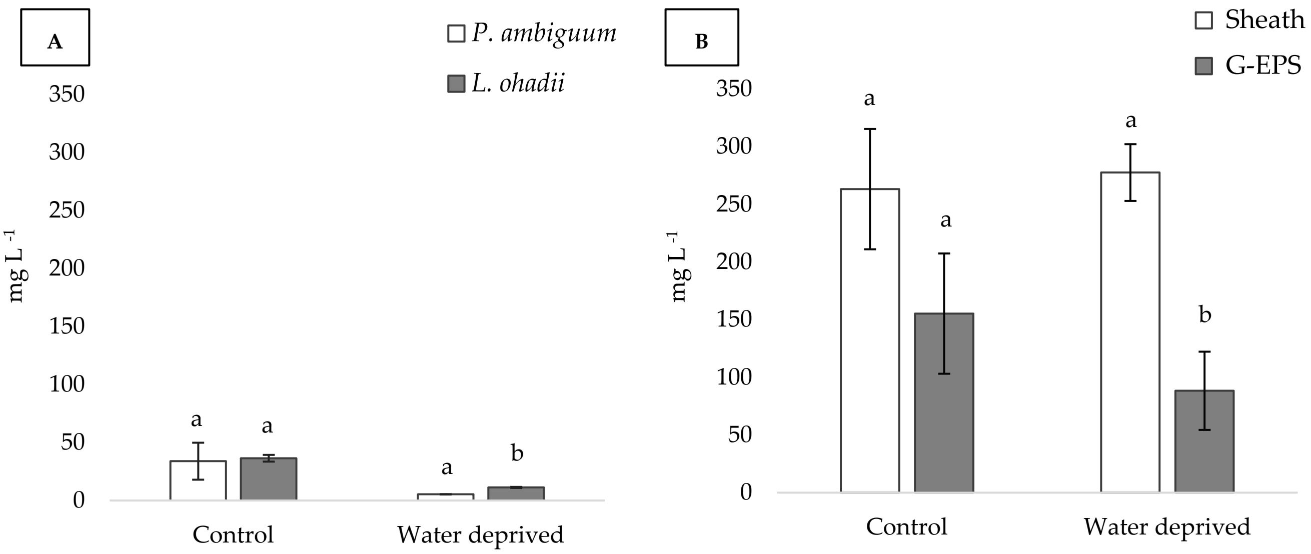

Figure S5. RPSs contents (A), sheath and G-EPS contents (B) in *P. ambiguum* and *L. ohadii* biofilms (values represent the mean of N = 3, error bars represent SD). Different letters represent significant differences ($P < 0.05$) in each condition.

Table S1. Diversity indices of the biocrusts EPS.

Culture	Sampling time	EPS fraction	Strain	Number of sugars	Dominance D	Shannon H	Equitability J
Biocrust	4 weeks watered	LB-EPS	*P. ambiguum*	6	0.30	1.35	0.75
			L. ohadii	8	0.39	1.27	0.61
	4 weeks watered	TB-EPS	*P. ambiguum*	8	0.36	1.33	0.64
			L. ohadii	10	0.51	1.11	0.48
	Control	LB-EPS	*P. ambiguum*	7	0.32	1.32	0.69
			L. ohadii	7	0.21	1.66	0.85
	Water deprived	LB-EPS	*P. ambiguum*	8	0.70	0.73	0.35
			L. ohadii	7	0.30	1.53	0.79
	Control	TB-EPS	*P. ambiguum*	6	0.42	1.20	0.67
			L. ohadii	9	0.44	1.27	0.58
	Water deprived	TB-EPS	*P. ambiguum*	6	0.48	1.00	0.53
			L. ohadii	9	0.45	1.20	0.55

Table S2. Diversity indices of the biofilms EPS.

Culture	Sample	EPS fraction	Strain	Number of sugars	Dominance D	Shannon H	Equitability J
Biofilm	4 weeks with medium	RPS	*P. ambiguum*	6	0.82	0.47	0.26
			L. ohadii	10	0.41	1.44	0.60
	4 weeks with medium	Sheath	*P. ambiguum*	11	0.60	1.02	0.42
		G-EPS	*L. ohadii*	7	0.55	1.03	0.50
	Control	RPS	*P. ambiguum*	9	0.41	1.23	0.56
			L. ohadii	10	0.18	2.04	0.85
	Water deprived	RPS	*P. ambiguum*	6	0.30	1.32	0.73
			L. ohadii	5	0.35	1.27	0.71
	Control	Sheath	*P. ambiguum*	10	0.57	1.08	0.46
		G-EPS	*L. ohadii*	10	0.49	1.21	0.50
	Water deprived	Sheath	*P. ambiguum*	11	0.75	0.73	0.30
		G-EPS	*L. ohadii*	8	0.51	1.10	0.50

Table S3. Sugar total mols and mols of each monosaccharide in each EPS fraction for *P. ambiguum* biocrusts.

	**LB-EPS 4 weeks**	**TB-EPS 4 weeks**	**LB-EPS control**	**TB-EPS control**	**LB-EPS water deprived**	**TB-EPS water deprived**
Total *	12,879	95,882	13,597	14,369	25,141	75,254
Fuc	0,073	0,427	0,071	0,188	0,045	0,282
Rha	0	0	0,122	0	1,094	0
GalN	0	0	0	0	0,069	0
Ara	0	0,084	0	0	0	0
GlcN	0	0,308	0	0	0,065	0,119
Gal	0	0,281	0	3,292	0,428	9,799
Glc	4,432	87,166	5,685	8,436	20,952	57,875
Man	0	0	0,000	0,000	0	0
Xyl	0,023	2,128	0,161	0,498	0	0
Fru	0	0	0	0	0	0
Rib	1,326	0	1,075	0	0	0
GalA	5,127	1,560	4,915	0,937	1,723	6,232
GlcA	1,897	3,928	1,569	1,018	0,766	0,947

* Numbers given in μmol.

Table S4. Sugar total mols and mols of each monosaccharide in each EPS fraction for *L. ohadii* biocrusts.

	**LB-EPS 4 weeks**	**TB-EPS 4 weeks**	**LB-EPS control**	**TB-EPS control**	**LB-EPS water deprived**	**TB-EPS water deprived**
Total *	19,349	24,387	7,915	19,553	30,936	9,607
Fuc	0,139	0,125	0,075	0,109	1,499	0,044
Rha	0,260	0,072	0,277	0,172	3,148	0,046
GalN	0	0	0	0	0	0
Ara	0,112	0,020	0	0	0	0,115
GlcN	0,196	0,075	0	0,032	0	0,152
Gal	1,654	0,872	1,095	2,804	5,054	1,442
Glc	9,315	17,267	1,819	12,786	17,006	6,281
Man	0	2,388	0	0,995	2,516	0
Xyl	0	0,476	0,866	0,970	0	0
Fru	0	0	0	0	0	0
Rib	0	0	0	0	0	0,041
GalA	6,745	0,714	2,049	0,721	1,054	0,666
GlcA	0,928	2,378	1,733	0,963	0,659	0,819

* Numbers given in µmol.

Table S5. Sugar total mols and mols of each monosaccharide in each EPS fraction for *P. ambiguum* biofilms.

	**RPS 4 weeks**	**Sheath 4 weeks**	**RPS control**	**Sheath control**	**RPS water deprived**	**Sheath water deprived**
Total *	18,029	71,338	17,328	149,102	9,153	121,146
Fuc	0,165	0,962	0,235	2,222	0,052	1,592
Rha	0,279	1,374	0,253	3,419	0	2,280
GalN	0	0	0	0	0	0
Ara	0,132	0,435	0,113	1,642	0,005	0,633
GlcN	0,000	0,387	0,151	1,573	0	0,657
Gal	0,000	6,727	0,191	12,463	0	3,297
Glc	16,289	54,917	10,704	111,424	3,283	104,956
Man	0	0	0,000	0,000	0	0
Xyl	0	2,143	0,374	8,297	0	3,179
Fru	0	0,924	0	1,877	0	1,371
Rib	0	0,225	0	0	0,638	1,101
GalA	0,273	2,456	4,202	4,813	2,992	0,994
GlcA	0,891	0,787	1,106	1,370	2,181	1,087

* Numbers given in µmol.

Table S6. Sugar total mols and mols of each monosaccharide in each EPS fraction for *L. ohadii* biofilms.

	RPS 4 weeks	G-EPS 4 weeks	RPS control	G-EPS control	RPS water deprived	G-EPS water deprived
Total *	51,966	34,434	23,410	145,192	5,867	51,175
Fuc	0,725	0,059	0,441	1,015	0,094	0,305
Rha	0,678	0,640	1,058	6,132	0	1,397
GalN	0	0	0	0	0	0,000
Ara	0,247	0	0	0,257	0	0,403
GlcN	0,336	0	0	0,116	0	0,115
Gal	3,465	3,947	1,461	12,598	0,075	8,015
Glc	34,331	26,733	9,484	109,589	1,589	38,111
Man	5,157	0	1,816	0	0	0
Xyl	3,301	0	2,317	0	0	0
Fru	0	0,290	0,206	9,966	0	0
Rib	0	0	0,892	0,840	0	0
GalA	0,211	0,861	3,378	3,143	3,216	1,114
GlcA	3,515	1,905	2,358	1,536	0,894	1,716

* Numbers given in μmol.
